# Vibrational communication and mating behavior of the greenhouse whitefly *Trialeurodes vaporariorum* (Westwood) (Hemiptera: Aleyrodidae)

**DOI:** 10.1038/s41598-021-85904-0

**Published:** 2021-03-22

**Authors:** Valeria Fattoruso, Gianfranco Anfora, Valerio Mazzoni

**Affiliations:** 1 Center Agriculture Food Environment, University of Trento, Via E. Mach 1, 38010 San Michele all’Adige, TN Italy; 2 Research and Innovation Centre, Fondazione Edmund Mach, Via E. Mach 1, 38010 San Michele all’Adige, TN Italy

**Keywords:** Animal behaviour, Entomology, Invasive species, Behavioural methods

## Abstract

The greenhouse whitefly (GW), Trialeurodes vaporariorum is considered one of the most harmful insect pests in greenhouses worldwide. The GW mating behavior has been partially investigated and its vibrational communication is only in part known. A deeper knowledge of its intraspecific communication is required to evaluate the applicability of control methods based on techniques of behavioral manipulation. In this study, for the first time, we provided a detailed ethogram of the GW mating behavior and we characterized the vibrational signals emitted during the process of pair formation. We characterized two types of male vibrational emissions (“chirp” and “pulses”), differently arranged according to the behavioral stage to form stage-specific signals, and a previously undescribed Male Rivalry Signal. We recorded and characterized two new female signals: The Female Responding Signal and the Female Rejective Signal. The mating behavior of GW can be divided into six different stages that we named “call”, “alternated duet”, “courtship”, “overlapped duet”, “mating”, “failed mating attempt”. The analysis performed with the Markovian behavioral transition matrix showed that the “courtship” is the key stage in which male exhibits its quality and can lead to the “overlapped duet” stage. The latter is strictly associated to the female acceptance and therefore it plays a crucial role to achieve mating success. Based on our findings, we consider the use of vibrational playbacks interfering with GW mating communication a promising option for pest control in greenhouses. We discuss the possibility to start a research program of behavioral manipulation to control the populations of GW.

## Introduction

Knowing the behavior of an insect pest is fundamental to develop an effective population control method based on a technique of behavioral manipulation^1^ which is a suitable strategy in the context of Integrated Pest Management (IPM) and organic farming^2–4^. Depending on the species, the behavior can be influenced by the perception of different cues such as visual, chemical and tactile stimuli, but also of acoustic and vibrational waves^5–7^. Communication through substrate-borne vibrations is known to be ancient and spread in many arthropods’ taxa, and thus it is considered as one of the most important communication modalities for understanding behavior^8,9^. In this respect, research about “applied biotremology” for insect pest control has been gaining more interest during the last few years. Examples of possible applications are trapping by releasing attractive signals, mating interference with masking noise, disturbance (i.e., feeding, oviposition) and repellence^2^. A clear example of it is the technique of mating disruption using vibrations which has been applied since 2017 in a commercial vineyard in Northern Italy, against the leafhopper *Scaphoideus titanus* Ball^10^. In another case, a vibration exciter made of magnetostrictive material has been used to control the population of the Japanese sawyer beetle *Monochamus alternatus* Hope^11^. These and other applications are showing to scientists and stakeholders that the concept of applied biotremology is not a mere hypothesis.

The object of this study is to provide evidence that the behavior of the greenhouse whitefly (GW), *Trialeurodes vaporariorum* (Westwood) (Homoptera: Aleyrodidae) can be potentially manipulated by means of a technique of applied biotremology. The GW is considered one of the most harmful and economically relevant insect pests in greenhouses worldwide, because it causes both direct, by subtracting nutrients during the feeding activity, and indirect damage, by transmitting viruses, to plants. In addition, the GW is highly polyphagous and it makes fruit unmarketable also by producing honeydew which generates a substrate for sooty molds that interfere with photosynthesis and reduces plant transpiration^12^.

In conventional farming, the principal control methods of the GW include the massive usage of synthetic insecticides^13^. In IPM and organic farming, other possible options include the use of essential oil extracts and of biological control agents. However, while the effects of the essential oils on the human health and on natural enemies are still controversial, the efficacy of the biological control agents is not always easy to achieve, depending upon numerous factors such as host plant quality, temperature, usage of fertilizer, dimension of the greenhouse, stage of infestation^14^. In this context, together with the increasing request of new environmentally sound approaches, the development of innovative techniques that do not rely on the use of chemicals, that can be easy to apply and with constant efficacy to control the GW, are largely demanded.

The mating behavior of GW has already been partially described, and in particular the courtship behavior was reported in different studies^15–17^. The courtship consists in a male that approaches and then courts a female while she is stationary during the extent of the courtship^18^. Females can mate several times but also reject males or interrupt the courtship by flicking the wings, grooming, or pivoting^15^. No receptive/rejecting acoustic signals were ever recorded. As for the male repertoire of vibrational signals, two vibrational elements have been described, both involved in the reproductive behavior: the chirp and the pulse, the latter emitted in series at regular repetition rate (pulse train)^17,19^. On the other hand, many important aspects of the mating behavior are still unclear. The role of vibrational signals in social interactions must be still described, especially when considering that GW lives in high density populations in which many individuals are in contact and share the same resources. Therefore, in this study we aim at investigating the intraspecific interactions of the GW in different social contexts, with focus to female acceptance and male-male competition. The latter was observed in a previous study without any mention of vibrational signals^15^. Our final goal is to provide a precise scheme of the GW mating behavior with the characterization of the associated vibrational signals and, thanks to this knowledge, to propose some possible approaches of behavioral manipulation to control this species.

## Materials and methods

### Insects rearing

The insects used for the experiment were obtained from a colony maintained at the Biobest company (Westerlo, Belgium) and shipped to the Fondazione Edmund Mach laboratory (San Michele all’ Adige, Trento, Italy). They have been reared in the greenhouse at 25 ± 2 °C, 70 ± 5% RH and 16:8 (L:D), in mesh cages (Bugdorm-6620, 60 × 60 × 120 cm, MegaView Science Co., Ltd., Taiwan) containing seedlings of tobacco (*Nicotiana tabacum*), tomato (*Solanum lycopersicum*), bean (*Phaseolus vulgaris*) and zucchini (*Cucurbita pepo*). All plants used for insect rearing were grown in the greenhouse at controlled conditions and no treatments were applied. Males and females were kept together in the same cages, then they were divided by gender at least 5 days before the experiment. In this way, we stimulated intersexual interactions in that individuals were neither just eclosed (i.e., too young) nor just mated. Our purpose was to describe the mating behaviour and associated vibrational signals in a colony where different types of individuals can meet and communicate.

### Experimental setup

The experiments were carried out in a sound insulated chamber in the laboratory of biotremology of the Fondazione Edmund Mach (Italy), from September 2014 to January 2015 and from October 2018 to February 2020. The insects were placed on a bean or tobacco leaf cut placed in a Petri dish (Ø 35 mm) positioned on an anti‐vibration table (Astel s.a.s., Ivrea, Italy). Insect behaviors were monitored via video surveillance (mod. HTC-TM700, Panasonic, Japan), to associate vibrational signals with corresponding behaviors. Vibrational signals produced by individuals were recorded using a laser Doppler vibrometer (PDV 100, Polytec, Germany). The laser vibrometer was focused on a small piece of reflective sticker placed on the leaf cut, to maximize the signal‐to‐noise ratio. The Petri dish had a hole on the top surface to let the laser beam pass through. Two softbox lights (50 × 70 cm) were used to illuminate the arena. The recordings were digitized using the software BK Connect (Brüel and Kjær Sound & Vibration A/S, Nærum, 104 Denmark) at 8.2 kHz sample rate and 24-bit depth resolution through a data acquisition device (LAN XI type 3050-B-040, Brüel and Kjær Sound & Vibration A/S, Nærum, Denmark), then they were stored onto a hard drive of a computer (HP, EliteBook 8460 p).

### Bioassays

Five different experiments were performed to observe the greenhouse whiteflies behavior and record the related vibrational signals.

1. Single Male (n = 37): one male was placed on the arena and recorded for 10 min to investigate the spontaneous calling activity.
2. Single Female (n = 30): one female was placed on the arena and recorded for 10 min to investigate the spontaneous calling activity.
3. Male Duos (n = 31): two males were placed on the arena and recorded for 10 min to investigate the possible occurrence of intrasexual communication.
4. Pairs (n = 157): one male and one female were placed on the arena and recorded to investigate the mating behavior and intersexual communication. In total, 30 min were given to the individuals to start the mating communication. The recording ended either after mating occurred or one of the individuals left the leaf.
5. Mixed Groups (n = 137): three males and two females were placed in the arena and recorded. In total, 30 min were given to the group to start the mating communication. The recording ended when one of the individuals left the leaf.

### Terminology and behavior characterization

The vibrational signals were identified and named according to previous literature and associated behavioral context.

The ‘pulse’ is defined as a physically unitary or homogeneous sound, composed of a brief succession of sine waves^20^ and the ‘pulse train’ as a succession of repetitive and temporally well-distinct group of pulses^21^. The ‘chirp’ is defined as a sequence of pulses having a bimodal phase in which an anterior pulse and a posterior pulse alternate^17^. As a ‘burst’, we defined a sudden and short vibrational signal.

For males tested individually and in duos (exp.1, 2, 3) the following parameters were recorded: time from the beginning of the trial to the emission of the first vibrational signal (call latency), number of individuals that emitted at least one signal during trials (signalling activity), number and type of signals emitted during the trial per individual.

As for insects tested in pairs (exp.4) we measured the following parameters: the first reply to a signal regardless of gender (latency to first duet) and the time between first duet and copula (latency to mating). For the analysis of the temporal sequence of different behaviors, a first-order Markovian behavioral transition matrix for the pair formation process was created using data of each couple. Data were taken from all insect pairs in which a mating attempt occurred regardless of whether it was successful or not (n = 46). Due to the absence of video recordings for 12 trials, only 34 were used to build the ethogram. Data were pooled in the analysis assuming no significant differences among individuals. Transition probabilities were calculated from the observed frequency of a transition between two events divided by the total number of occurrences of the first of the two events^22^. The following behaviors were selected to be used in the analysis: call, alternated duet, courtship, overlapped duet, unsuccessful mating, and mating. Expected values were calculated using the iterative proportional fitting method of Goodman (1968), then G-test (Williams’ corrected) was performed to determine the significance, after Bonferroni method, of the overall table and of transitions by collapsing the table in a 2 × 2 matrix^23^.

### Signal characterization

Spectral and temporal parameters of signals were analyzed with the software Raven Pro 1.6 (The Cornell Lab of Ornithology, Ithaca, NY, USA) using fast Fourier transform (FFT) type Hann with window length of 2048 samples and 75% overlap. The following parameters were measured for each signal: duration, fundamental frequency, and pulse repetition time (measured as the distance between the onset of two consecutive elements). The following type and number of recordings were used to characterize vibrational signals of the greenhouse whitefly: single males (n = 37), male duos (n = 32), pairs that mated (n = 34) and that did not (n = 10), and mixed groups that displayed male rivalry behavior and/or female rejection (n = 26). The mean value from three to five randomly chosen signals per type from each individual was used for the statistical analysis. Non-parametric tests were used when appropriate; all tests were two-tailed. The Kruskal–Wallis test followed by the Mann–Whitney pairwise test was used to compare the male signals (chirp and PT) duration and fundamental frequency, and the PT pulse repetition time across three different stages (i.e. call, alternated duet, courtship). The Mann–Whitney U-test was used to compare the call latency and the number of pulses and chirps between males in single and duos tests, while the t-test (unpaired) was used to compare the fundamental frequency and signal (chirp and PT) duration. The Mann–Whitney U test, again, was used to compare the fundamental frequency between chirp and MRS signal and between FRS and FRjS.

## Results

### Vibrational signals

**Figure 1:**
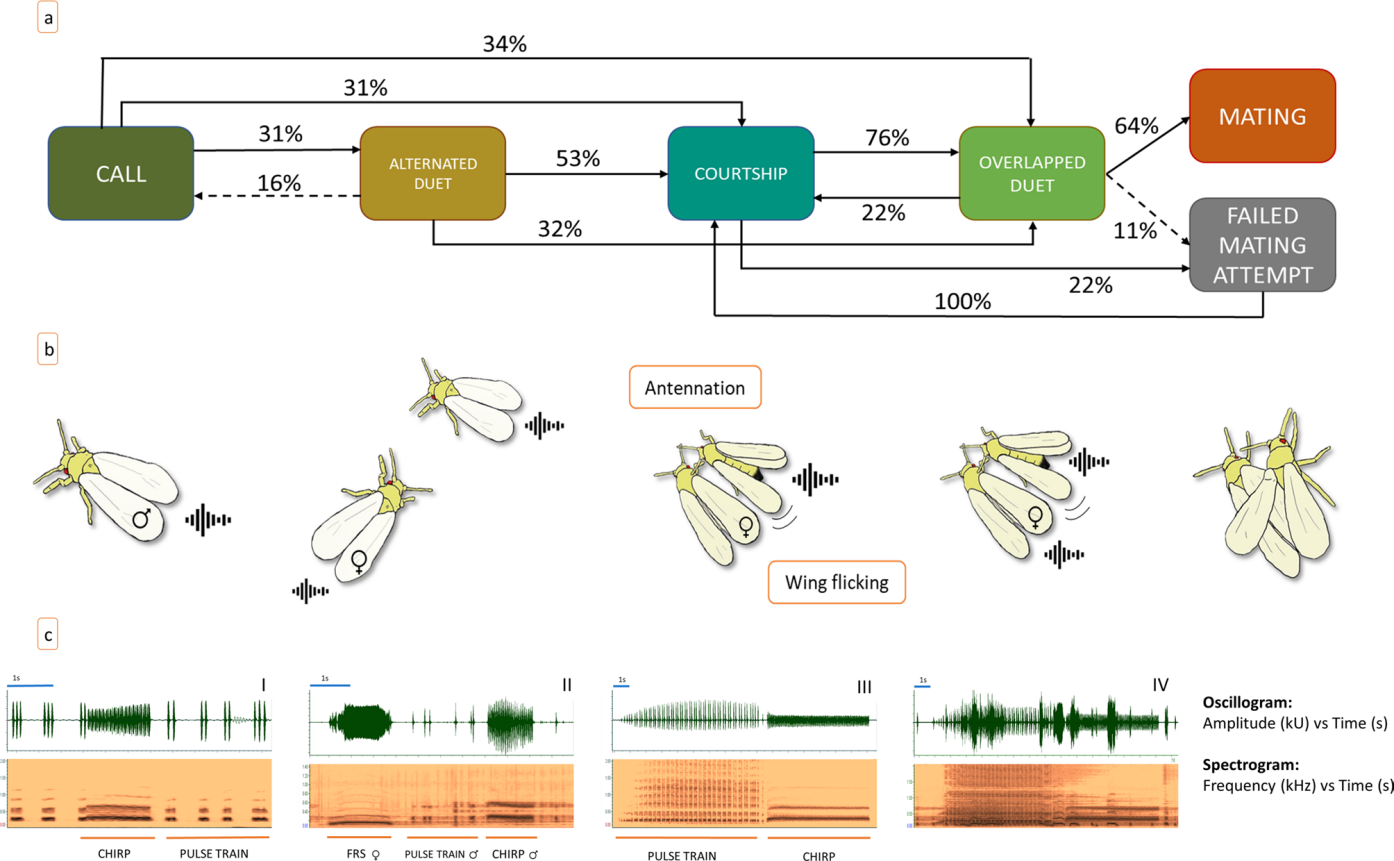
(**a**) Ethogram describing transition probabilities between behaviours that compose the process of pair formation in the greenhouse whitefly. Dashed lines represent no significant transitions (*P* > 0.05), whereas solid lines are significant transitions (*P* < 0.05). The percentage of observed transitions is indicated over each line. No significant transitions with percentages < 10% were not included in the ethogram; (**b**) A sequence of stereotyped mating behaviour in the greenhouse whitefly (author Sabina Avosani); (**c**) Oscillogram (above) and spectrogram (below) of GW vibrational signals. In I) male chirp and PT in call stage, II) male chirp and PT followed by FRS in alternated duet stage, III) male chirp and PT in courtship stage, IV) male chirp and PT overlapped by FRS in overlapped duet.

Males emitted three types of signals: the chirp, the pulse train (PT) and the male rivalry signal (MRS); females emitted two types: the female responding signal (FRS) and the female rejective signal (FRjS) (Figs. 1c,3). The signal production was associated with a slightly visible dorsoventral abdominal oscillation.

*Male signals.* The characteristics of the male signals are reported in Table 1. Chirp and PT are signals with a harmonic spectrum that were produced by males alone and in social interactions. Both signals are composed of a series of pulses of similar duration across the different stages, although they can considerably vary in terms of fundamental frequency, pulse repetition time and total signal duration. Such values can also vary according to the behavioral context in which they are emitted.

**Table 1:** Temporal and spectral parameters of GW male signals.

	Duration (s)			Frequency (Hz)			Pulse repetition time (s)		
	CALL	A. D.	COURTSHIP	CALL	A. D.	COURTSHIP	CALL	A. D.	COURTSHIP
Chirp	1.4 ± 0.2	1.5 ± 0.4	9 ± 2.7	257 ± 30	290. ± 34	307 ± 56	0.05 ± 0.003	0.05 ± 0.005	0.05 ± 0.004
Single Pulse	0.05 ± 0.01	0.07 ± 0.02	0.07 ± 0.01	248 ± 78	251 ± 27	239 ± 57	NA	NA	NA
Train of Pulses	10.6 ± 11.2	4.7 ± 2.3	10.4 ± 2.2	258 ± 25	257 ± 50	227 ± 40	4.9 ± 9.3	1.1 ± 1.3	0.2 ± 0.03
MRS	NA	NA	0.6 ± 0.3	NA	NA	327 ± 69	NA	NA	NA
FRS	NA	1.4 ± 1.5	NA	NA	161 ± 23	NA	NA	NA	NA
FRjS	NA	NA	0.4 ± 0.2	NA	NA	277 ± 71	NA	NA	NA

Means with standard deviation (SD) are shown.

*A.D.* alternated duet, *NA* not applicable.

The chirp is composed of two alternating elements emitted at rather constant pulse repetition time (Table 1): a short pulse (mean duration ± SD: 22 ± 4 ms) followed by a long pulse (40 ± 5 ms). On the contrary, the PT is composed of a series of single pulses of similar duration but rather variable repetition time (Table 1). From the comparison of the signals features taken from the exp.1 (single males) and exp.3 (male duos) (see below) we found that the fundamental frequency of chirp and PT signals was significantly higher in males duos than in single male experiments (chirp, t-test: t = 2.30, *P* = 0.02) (PT, t-test: t = 3.41, *P* < 0.001). We also observed a longer duration of the chirp signal in male duos (chirp t-test: t = 4.19, *P* < 0.001) (Table 2).

**Table 2:** Number of signals per individual and temporal and spectral parameters of GW male signals in exp.1 and exp.3.

	n. of signals		Duration (s ± SD)		Frequency (Hz ± SD)	
	1 M	2 M	1 M	2 M	1 M	2 M
CHIRP	3 ± 3	3 ± 4	1.43 ± 0.26	1.18 ± 0.32	257 ± 30	272 ± 43
PULSES	5 ± 9	9 ± 23	0.05 ± 0.01	0.05 ± 0.02	228 ± 35	282 ± 34

Means (± SD) are shown.

**Figure 2:**
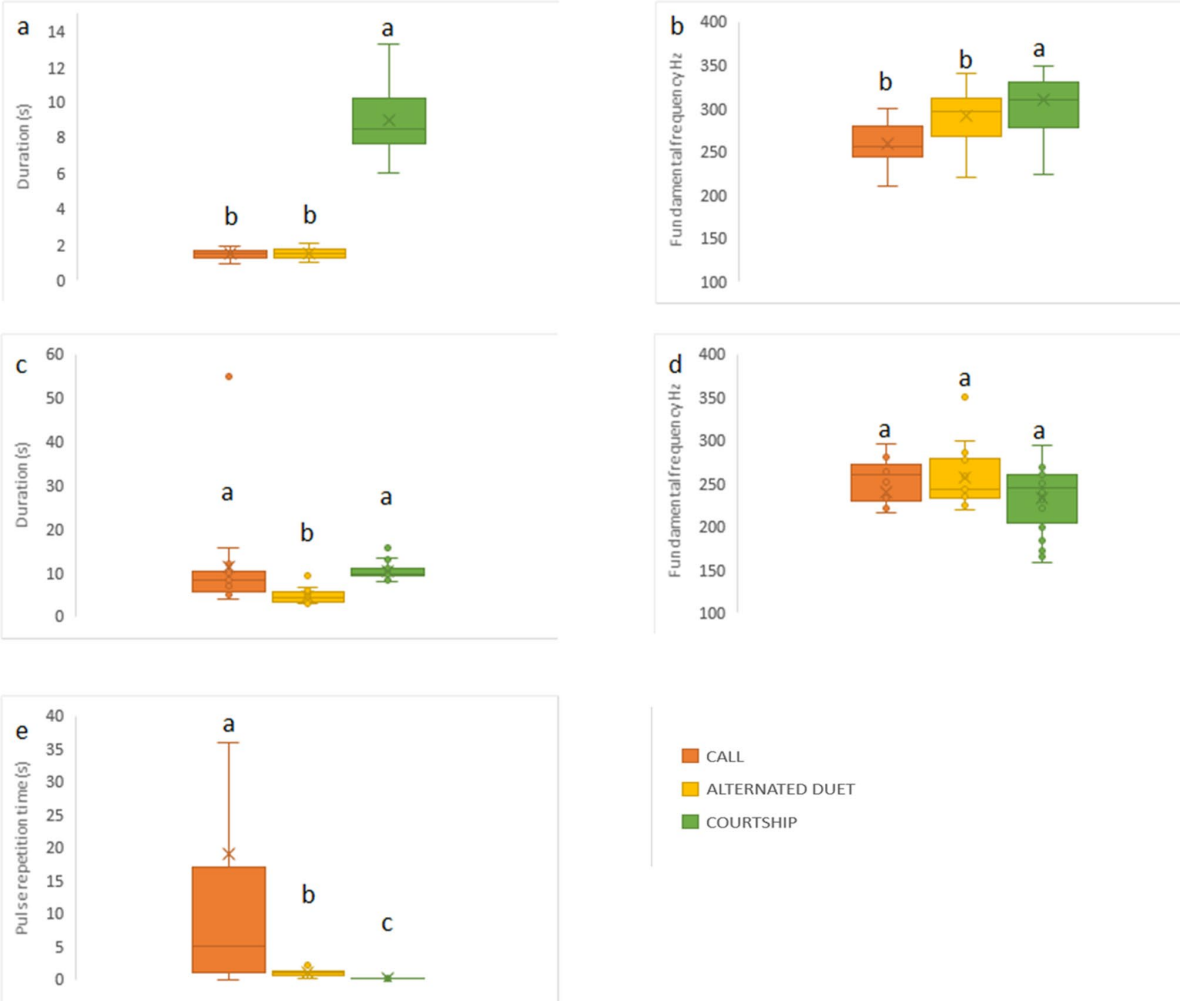
Boxplots of spectral and temporal parameters of GW vibrational signals across three different mating stages (exp. 4, pairs). In orange “call”, in yellow “alternated duet”, in green “courtship”. (**a**) chirp duration, (**b**) chirp fundamental frequency, (**c**) PT duration, (**d**) PT fundamental frequency, (**e**) pulse repetition time. Significant letters indicate significant differences among signals after Kruskal–Wallis test followed by Mann–Whitney pairwise test (*P* < 0.05).

The analysis of the signals emitted in exp.4 (pairs) showed a significant difference across the three different behavioral stages (i.e. “call”, “alternated duet” and “courtship”, see below, the behavioral analysis) in terms of chirp duration (Kruskall-Wallis: H = 48.78, *P* < 0.001) and fundamental frequency (Kruskal–Wallis: H = 18.8, *P* < 0.001) (Fig. 2a,b). We also observed significant differences in PT duration (Kruskal–Wallis: H = 27.2 *P* < 0.001) and PT pulse repetition time (Kruskal–Wallis: H = 31.25 *P* < 0.001) (Fig. 2c,e), while the PT fundamental frequency did not differ among the different stages (Kruskal–Wallis; H = 4.45, *P* = 0.10) (Fig. 2d).

As for the MRS signal, it is composed of a series of pulses almost fused together that are characterized by a fundamental frequency significantly higher than that of the chirp produced during the courtship (Mann–Whitney U test, Bonferroni corrected; U = 270, *P* < 0.001) (Table 1).

*Female signals.* The Female Responding Signal (FRS) has been observed in response to the male call during the “alternated duet” and “overlapped duet” stages. It is characterized by a burst with a variable duration and a mean fundamental frequency of 161 Hz (Table 1). As for the Female Rejective Signal (FRjS), it is composed of a series of short bursts significantly shorter than the FRS (t test: t:4.5 *P* < 0.001) and with a significantly higher fundamental frequency (Mann–Whitney U test Bonferroni corrected: U = 15, *P* < 0.001) (Table 1).

### Behavioral analysis

#### Exp. 1: single male

When placed alone on the leaf, 72% of males (N = 37) spontaneously emitted vibrational signals. All the active insects emitted at least one chirp and 74% (N = 27) emitted at least one pulse. Males emitted both chirps and pulses in different amounts (Table 2), with a variable call latency (73 ± 130 s).

#### Exp. 2: single female

When placed alone on the leaf, none of the females (n = 30) emitted spontaneous vibrational signals.

#### Exp. 3: male duos

When two males were placed together on the leaf, in 90% (N = 31) of the trials at least one male emitted a vibrational signal, although due to the small size of the arena it was not possible to establish whether one or both males where signalling. In 92% (N = 28) of the trials with signal emissions, at least one chirp was recorded. The mean number of chirps and pulses emitted per individual in each trial did not differ from the exp.1 (chirp, Mann–Whitney U-test: U = 320, *P* = 0.33; pulses, Mann–Whitney U-test: U = 265 *P* = 0.08) (Table 1). The mean (± SD) latency to the first signal was 79 ± 126 s.

#### Exp. 4: pairs and mating behavior analysis

When a male and a female were placed together on a leaf, 76% of males (N = 157) were active (i.e., they produced at least one vibrational signal) and 35% of females responded with a Female Responding Signal (FRS), thus establishing a duet with the male. 23% of pairs achieved mating, while we observed 6% of unsuccessful mating attempts. Latency to duet (mean ± SD 620 s ± 847) and to mating (mean ± SD 1390 ± 870 s) (N = 34) were rather variable.

We ideally divided the GW mating behavior in four different stages each characterized by stereotyped actions of males and females (Online Resource 1):

1. *Call*: The male emits spontaneous call signals (chirp and pulses).
2. *Alternated duet*: The female responds to the male calling signals with the FRS and establishes a duet with him. The individuals are relatively distant (more than one body length), and the male actively searches for the female.
3. *Courtship*: The male is now at short distance from the female, standing by her side, while keeping on emitting chirps and PT. At this stage, the male circles the female several times, touching her antennae and forelegs with his forelegs. Eventually, the male takes place alongside the female with an angle of about 30°. During this action, the female is mute.
4. *overlapped duet*: The female replies to the male signals (chirp and PT) by overlapping her own signal (FRS) with his.

The behavioral analysis based on the Markovian transition matrix (Fig. 1a,b) can be described in these terms: the male always called first, by alternating chirp and PT, and the female by responding with the FRS (2) established the alternated duet (31%) during which the male searched for her. When a male reached the female, the courtship stage (3) began (53%). In some cases, however, males after calling reached a silent (i.e., not responding) female and then started the courtship (3) (31%). In addition, a number of males (34%) went to the overlapped duet directly (4). This happened when a male localized a female who immediately responded to the male signals. In 32% of the cases the male skipped directly from the alternated to the overlapped duet stage (4) (thus bypassing the courtship). This happened when a male approached a female who did not stop responding to his signals once he got close to her.

During the courtship phase, if the female did not show any rejective behavior (see below results of exp.5), we observed three contemporary actions: antennal rubbing, wing flicking and emission of PT and chirp. These actions were repeated five or more times. In detail: a male circles the female several times, touching her antennae and forelegs with his forelegs then, he takes place alongside the female with an angle of about 30° and he rubs with his antennae the female’s closest antenna and pronotum. At the same time, he performs a persistent wing flicking, accompanied by a long PT (mean ± SD 265 ± 153 s). During this, the male emits alternatively PT and chirps, where the PT is always associated with wing flicking, whereas the female remains silent and often did not interrupt ongoing activities (i.e., feeding or grooming). Some males (22%) attempted mating before the female responded and all of them failed. Instead, males that performed the overlapped duet, which started when the female replied by overlapping the male’s with her own signal (76%), most of the times succeeded in mating (64%). Only in few trials (11%), the female rejected the male after the performance of the overlapped duet. When this happened, the males resumed from the courtship (Online resource 1).

#### Exp 5: mixed groups

**Figure 3:**
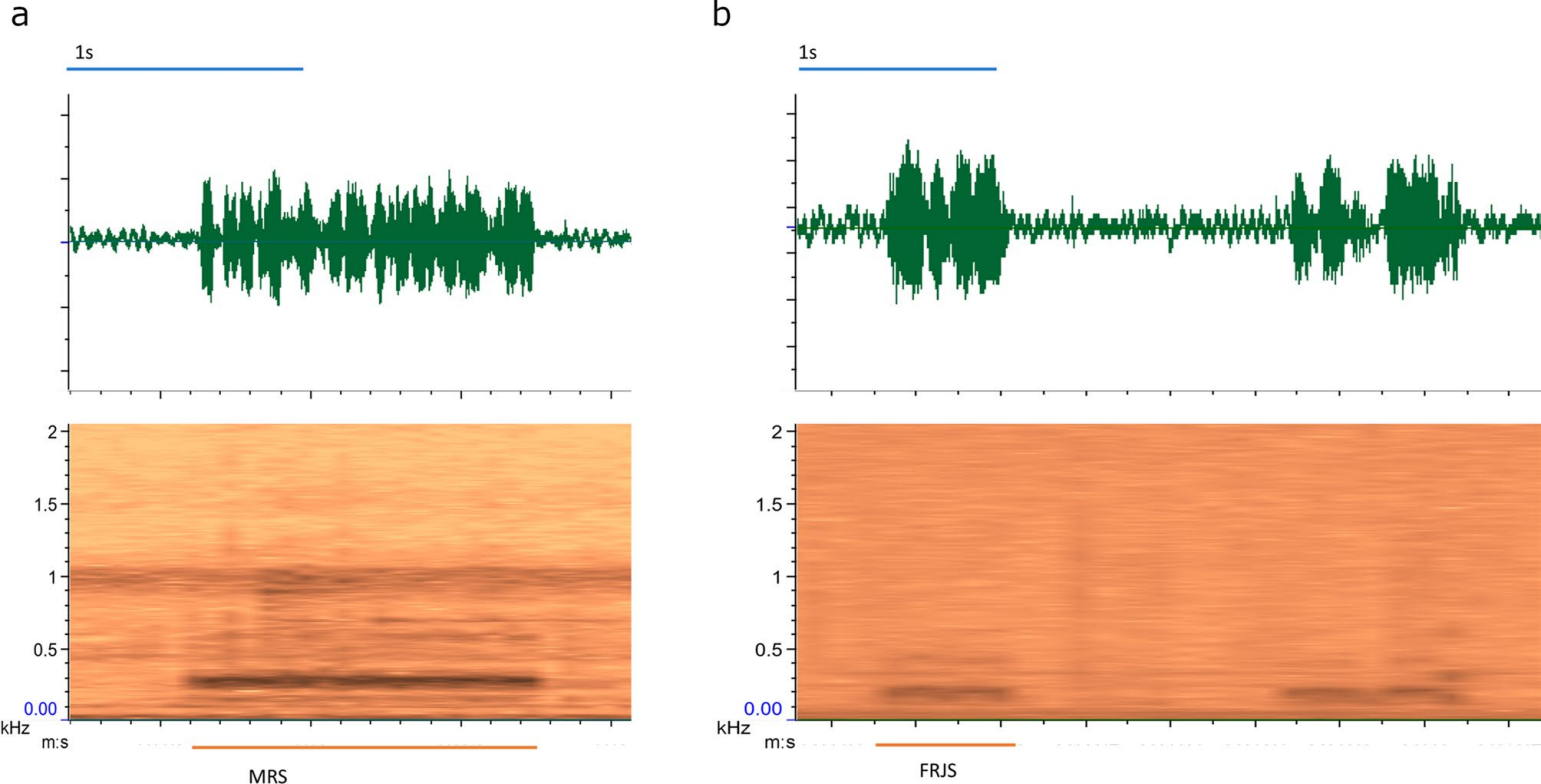
Oscillogram (above) and spectrogram (below) of GW vibrational signals. In (**a**) MRS, (**b**) FRjS.

In Exp. 5 (groups of 3 males and 2 females), we observed the occurrence of male rivalry behavior and female rejection behavior, in 11% and 17% of the trials (N = 137), respectively. The male rivalry behavior occurred in different contexts and was characterized by the emission of a specific Male Rivalry Signal (MRS) (Fig. 3b). In three recordings it was observed when a male passed close by another male; in this case, the approaching male was turned away by the first one via MRS emission paired with wing flicking; in three other replicates, the male, after mating, remained in the female proximity while producing the MRS signal. In most of the trials where the rivalry behavior was observed (60%, N = 15), a disruptive male approached a couple during the courtship (Online resource 2). The disruptive male walked either between the male and female or next to the female. At this point, a male-male competition started: both males attempted to copulate with the female by protruding the aedeagus to seize her genitalia. At the same time, they physically competed by spreading and flapping the wings against the rival while emitting MRS. In all these trials however, none of the males achieved the copulation and the female eventually walked away.

The female rejective behavior occurred in different modalities (Table 3): the female either walked away during the courtship or repelled the approaching male by wing flicking; in other cases, we observed the emission of a specific Female Rejective Signal (FRjS) (Fig. 3a) that in some cases was associated with a persistent wing flicking (Online resource 3). After the FRjS emission, the female either walked away or parried the male’s aedeagus with the legs when he attempted to copulate. In most of the cases the female rejected the male at the early stage of the courtship (i.e. during the male circling action), except for when the female walked away or parried male’s aedeagus which happened at the end of the courtship (i.e. when the male was positioned alongside the female with an angle of 30° and was alternating chirp and PT).

**Table 3:** Behaviors characterizing the female rejection.

Modality	n	%	Context
Walk away	9	37	Late courtship
FRjS	3	12	Early courtship
Wing flicking	4	16	Early/late courtship
Wing flicking + FRjS	3	12	Early/late courtship
FRjS + walked away	2	8	Early/late courtship
Legs parring	3	12	Late courtship

## Discussion

In this study, we gave a comprehensive description of the mating behavior of the greenhouse whitefly, *T. vaporariorum*. In particular, we defined the strict association between vibrational signals and behavioral steps of the pair formation process, from the male call to the final mating. We also described some social interactions between two or more individuals of both sexes, confined to a small portion of leaf, thus simulating a natural occurring aggregation. In this regard, we found that males tend to modify the quality of their vibrational signals, by changing some spectral features, according to either the social context or the behavioral step. For example, they tend to increase the fundamental frequency of their signals (i.e., chirps and PT) when in the presence of potential rivals. A possible explanation of this behavior could be associated with the male competition for food and/or mating. In fact, species that live in high population densities are subjected to strong male-male competitions and a male needs to show his quality to females but also to be clearly recognizable from the others^24^. The higher quality can be witnessed by the emission of specific aggressive calls which are characterized by lower frequencies, like in some anurans^25^ or in Chiropteran where the relative frequency of the social calls increases when more individuals compete for a food source. An example of individual recognition behavior is the change of frequency of the calling song to avoid signal overlapping thus allowing an individual to perceive the presence of more potential partners. Frequency overlapping, in general, can be noxious to animal communication, and male responsiveness can be reduced when background noise from conspecific signals obscure the species-specific temporal pattern of a female song^26^. In the southern green stink bug, *Nezara viridula* (Heteroptera, Pentatomidae), females were found to change their calling song frequency to let the males recognize them when exposed to a disturbance stimulus^27^. Even if small variations of the frequency pattern may potentially affect the partner responsiveness to a call^28^, overlapping frequencies can seriously compromise the signal reception^29^. In this way, the change to a different value of frequency in presence of other calling males seems to be a more desirable solution.

Another signal variation that we observed in GW males, in the presence of another male (i.e., male duos trials), regarded the chirp duration. By increasing the duration of a mating signal, some species also increase the chances to elicit the female response at the earliest stage of the mating behavior^30^. In various acoustic insects, females prefer longer calls and males can vary their length by adding or subtracting call elements^31^. However, a limit of our study was that we could not associate the signal emissions to specific individuals, therefore we did not determine not only if one or both the males were actually singing but also whether this change of chirp duration involved one or both the individuals. A definitive explanation about male-male calling interactions and how males regulate their calling activities should be provided with additional experiments with the use of playbacks to stimulate single specimens.

In general, we need to consider that the alteration of the signal features is a common strategy in animals with a complex mating behavior in which different stages can alternate in a non-linear sequence^29,32,33^. Such an intricate behavior is on the one hand, at the basis of a species-specific mate recognition system, on the other hand, is a result of the sexual selection that worked to shape signals with certain characteristics that are able to elicit the female acceptance to mate^34^. Despite the considerable knowledge about vibrational signal production in the family Aleyrodidae^19^, we still have little information about the importance of the courtship and of the female choice in driving the reproductive isolation and speciation in this family. Aleyrodid species are known to be morphologically similar and to form a species complex (i.e. *Bemisia tabaci*) with several biotypes^35^, where the characterization of the mating behavior can be an important tool to discriminate among them. For instance, variations in the courtship behavior between different *B. tabaci* biotypes demonstrated the presence of pre-copulation barriers^36,37^. Moreover, the analysis of male vibrational signals during the courtship, combined with genetic and morphological analysis, allowed to discriminate between the camellia spiny whitefly *Aleurocanthus camelliae* and the citrus spiny whitefly *Aleurocanthus spiniferus*^18^. In such a context, knowing the characteristics of the mating ritual may lead to distinguish, not only among different species, but also among different populations. For example, before this study, the GW mating behavior was described only from Japanese populations where the pair formation process started with the male approaching a female before emitting any vibrational signal (i.e. courtship stage)^17^. Instead, in our study with European populations of GW, we observed that the male, before starting the approach, emits calling signals which can elicit the female response from a certain distance. Such a difference between geographically distant GW populations seems to suggest a different strategies of mating behavior, likely associated to distinct populations or biotypes. On this regard, it would be interesting to test them with crossed mating trials (Japanese vs Europeans) to assess the effects of the observed differences on the mating success rate.

In our study, we also measured a difference of male signal parameters between different behavioral phases of the pair formation process and in particular between the courtship stage and the call and alternated duet stages. We found a significant increase of signal duration, fundamental frequency and pulse repetition rate. The duration of the courtship stage was very variable in our trials, from zero (it was skipped when females replied immediately to the male signals) up to 78 min. This means, in first instance, that the role of the courtship is to elicit the female response and thus promoting her acceptance to mate. Indeed any single behavioral step is functional to elicit the female’s acceptance and in fact, whenever females showed high responsiveness since the early stage of the mating process, males could skip whole stages and even go directly from the call to the final precopula stage, the alternated duet. It also indicates that males are available to spend a remarkable amount of energy to perform the courtship^38^. The use of elaborated and energetic signals during the courtship is rather common in animals^34^. For example, the leafhopper *S. titanus* and the glassy-winged sharpshooter *Homalodisca vitripennis* have a mating strategy that reminds the GW’s, starting with a call which is followed by the location of the partner and by the courtship. While during call and location males make use of extremely simplified signals, during the courtship they emit the most elaborated (and energetically demanding) signals, through which they try to convince the female to accept the mating^21,39^. A study of Las (1980) demonstrated that the GW courtship persistence (i.e., duration) is an important trigger to address the female choice. A fast and prolonged male “cycling rate” (alternation of wing flicking and antennation) during the courtship is preferred by females who become even more selective after the first mating. On the other hand, in our tests, males showed a remarkable perseverance in courting the females. The ethogram showed that after a failed mating attempt, a male always restarted from the courtship. This means that the courtship phase is the key part of the mating process but also that the female choice drives the selection in favor of “stubborn” males that persist in courting the potential partner, performing a prolonged courtship, even if the first mating attempt fails. Stubbornness affects male’s survival for its energetic cost and risk of eavesdropping. Such character fits the handicap theory model, in which condition dependent and costly traits are honest indicators of male quality^40,41^. On the other hand, the option of an easy surrender, and the search for another available female, after investing so many energies in courting the first one, seems to be not convenient for the male in that it would mean to spend more energy in searching for/courting a new partner also risking the possibility of dealing with competitors^42^.

In the GW, the male courtship can be considered successful when the mating moves to the overlapped duet stage in which the female emits the Female Responding Signal (FRS). The FRS is produced in synchronous with the courtship chirp and PT and, for this reason, it requires high degree of coordination between male and female. The presence of female acceptance signals synchronized with the male’s is known for the whitefly species *Aleurothrixus floccosus* (Maskell)^43^, in which the female signal can partially overlap the male’s one, but it was unknown in the GW, until now.

Another signal that we found for the first time in the GW is the male rivalry signal (MRS). Males exhibit aggressiveness towards other males. A random encounter on the leaf is enough to trigger the expression of rivalry behavior in presence of a female. Such interaction has never been observed in duos, but only in groups with responsive females, thus suggesting that the presence of receptive/active females is required to trigger the MRS production and thus provoking a context of aggressiveness and competition between males. Another male rivalry behavior that we observed in the presence of a receptive female is the silent approach (satellite behavior) to intercept a female while duetting with another male^44^. This behavior is known in other aleyrodids like in *B. tabaci*. In this species, rival males interrupt the ongoing courtship of the duetting male by approaching the female from the opposite side. In response to the competitor, the first male spreads the wings and beats the rival on the head^45^. In GW, the rivalry behavior is associated with the continuous production of the MRS, which is the male signal at highest frequency. Such finding strengthens the hypothesis that the frequency shift has a role in competitor’s deterrence. The rivalry behavior of GW seems to be extraordinarily strong, as much to push females to abandon the interaction with both males. In our experiments, none of the females, even those that had already established a duet with a male, eventually mated. On the contrary, they left the arena before the end of the trial. Our findings are consistent with previous observations of GW behavior, in which the contended female always walked away when two males were competing^15^. Therefore, we can speculate that the adaptive advantage of the male rivalry behavior in GW is not immediate and the disruption of another male’s attempt could provide more chances in the future to the intruder, by leaving a receptive female unmated. Beside the effects of the male’s rivalry, we also observed females that refused to mate and rejected approaching males with the emission of specific vibrational signals. There are several reasons to refuse mating: immature females are not yet available to mate, and recently mated females must undergo to a refractory period before they accomplish other copulations^15^. On the other hand, a mature female can choose whether to accept or not a courting male depending on the level of his fitness which is, very likely, testified by the courtship performance. Females can evaluate the male’s quality based on the courtship persistence, so that they need to let males perform the whole ritual before choosing whether to mate or not^46^. In fact, we observed both females that rejected approaching males and females that rejected them at the end of the courtship performance. The latter, in particular, was associated to wing flicking and/or male’s aedeagus parrying with the legs. Similar behaviors were also observed in *B. tabaci*, in which the female can either walk or fly away from approaching males, flap the wings or push the male’s abdomen away with the middle pair of legs^45^. What seems to be a peculiar treat of GW is the use of a specific rejective signal (FRjS). The emission of FRjS seems to reinforce the motivation of the female to reject the male. However, it is not clear to us why the FRjS signal has been observed only in the group (males and females together) trials and never in pairs (one male and one female). Our hypothesis is that in case of groups, males can approach the “wrong” female, who was close the receptive one. This implies that males are not capable of precisely locating the responding female and that the emission of FRjS by an unreceptive female would help the males to not waste too much time (and energy) with them.

To conclude, this study unveiled many aspects of the mating behavior of the GW that were previously overlooked and thus it contributes to fill several gaps of knowledge that will be important to start a program in the field of applied biotremology^10^. The question, from which originally arose this research study, was whether the use of vibrational signals could be suitable to manipulate the mating behavior of the GW. We can say that the vibrational communication is fundamental to accomplish mating and, in our trials, with pairs and groups, we never observed mating without the exchange of vibrational signals between male and female. This means that the interruption or the disruption of this communication could be potentially useful to reduce the rate of mating success. Manipulation of intraspecific communication by means of vibrational signals has been already developed for other insect species both in the lab and in the field^10^. For example, the male rivalry signal has been exploited for the development of a vibrational mating disruption strategy against the grapevine leafhopper *Scaphoideus titanus*^29^, while the female playback has been used to attract and trap males in the brown marmorated stink bug *Halyomorpha halys*^47^. The use of playbacks that cover the fundamental frequencies of the male and female signals could be used to mask their communication^2^. Another possible approach could be to use signals that mimic the natural signals of the species^48^. In the case of the GW, the FRS could be employed to disrupt males and induce them in courting unreceptive females. This would lead to a substantial reduction of the mating success rate but also to a considerable increase of wasted energy caused by the male persistence in courting unreceptive females. Another possible outcome could be a change of the gender balance in the population. GW females reproduce by arrhenotokous parthenogenesis in which unfertilized eggs develop into males^49^. Delays in mating could lead to a sex bias that could eventually mine the population structure. Another option is the use of the MRS to generate an aggressive and stressful environment. The transmission of MRS into the plant tissues in loop could eventually negatively affect the development of GW populations. All these approaches are potentially effective and could be in the future considered as tools for IPM and/or organic protection programs. Further applied research will provide a final answer to our question and will test the effectiveness of behavioural manipulation strategies for the control of the GW. Finally, considering that the GW uses a short range sexual pheromone emitted by females^50^ olfactory and vibratory cues could be potentially integrated to develop new pest control technologies^10^.

## Supplementary Information

Supplementary Video 1.

Supplementary Video 2.

Supplementary Video 3.
